# Antigen Sampling *CSF1R*-Expressing Epithelial Cells Are the Functional Equivalents of Mammalian M Cells in the Avian Follicle-Associated Epithelium

**DOI:** 10.3389/fimmu.2019.02495

**Published:** 2019-10-22

**Authors:** Adam Balic, Cosmin Chintoan-Uta, Prerna Vohra, Kate M. Sutton, Robin L. Cassady-Cain, Tuan Hu, David S. Donaldson, Mark P. Stevens, Neil A. Mabbott, David A. Hume, Helen M. Sang, Lonneke Vervelde

**Affiliations:** ^1^Division of Developmental Biology, The Roslin Institute, University of Edinburgh, Edinburgh, United Kingdom; ^2^Division of Infection and Immunity, The Roslin Institute, University of Edinburgh, Easter Bush, Edinburgh, United Kingdom; ^3^Division of Genetics and Genomics, The Roslin Institute, University of Edinburgh, Easter Bush, United Kingdom

**Keywords:** mucosa-associated lymphoid tissue, antigen uptake, M cell, CSF1R, avian, *Salmonella*, transgenic reporter chicken, lectin

## Abstract

The follicle-associated epithelium (FAE) is a specialized structure that samples luminal antigens and transports them into mucosa-associated lymphoid tissues (MALT). In mammals, transcytosis of antigens across the gut epithelium is performed by a subset of FAE cells known as M cells. Here we show that colony-stimulating factor 1 receptor (CSF1R) is expressed by a subset of cells in the avian bursa of Fabricius FAE. Expression was initially detected using a *CSF1R*-reporter transgene that also label subsets of bursal macrophages. Immunohistochemical detection using a specific monoclonal antibody confirmed abundant expression of CSF1R on the basolateral membrane of FAE cells. *CSF1R*-transgene expressing bursal FAE cells were enriched for expression of markers previously reported as putative M cell markers, including annexin A10 and CD44. They were further distinguished from a population of *CSF1R*-transgene negative epithelial cells within FAE by high apical F-actin expression and differential staining with the lectins jacalin, PHA-L and SNA. Bursal FAE cells that express the *CSF1R*-reporter transgene were responsible for the bulk of FAE transcytosis of labeled microparticles in the size range 0.02–0.1 μm. Unlike mammalian M cells, they did not readily take up larger bacterial sized microparticles (0.5 μm). Their role in uptake of bacteria was tested using *Salmonella*, which can enter via M cells in mammals. Labeled *Salmonella enterica* serovar Typhimurium entered bursal tissue via the FAE. Entry was partially dependent upon Type III secretion system-1. However, the majority of invading bacteria were localized to *CSF1R*-negative FAE cells and in resident phagocytes that express the phosphatidylserine receptor TIM4. *CSF1R*-expressing FAE cells in infected follicles showed evidence of cell death and shedding into the bursal lumen. In mammals, CSF1R expression in the gut is restricted to macrophages which only indirectly control M cell differentiation. The novel expression of CSF1R in birds suggests that these functional equivalents to mammalian M cells may have different ontological origins and their development and function are likely to be regulated by different growth factors.

## Introduction

The mucosal immune system in the gut is continuously exposed to foreign material in the form of food antigens, commensal organisms, and potential pathogens. To initiate an effective mucosal immune response, antigens are transported across the epithelium into MALT. In birds, which lack draining lymph nodes, MALT are the main sites of antigen-specific activation of mucosal B and T cells. In the chicken gut, the MALT are comprised of solitary or aggregated lymphoid follicles with structures resembling mammalian Peyer's patches (PP) as well as avian-specific lymphoid tissues such as bursa of Fabricius, caecal tonsils and Meckel's diverticulum (1). In both mammals and birds the specialized follicle-associated epithelium (FAE) overlying the MALT contains a population of specialized highly endocytic cells that transfer particulate antigens and microorganisms to underlying immune cells (2). In mammals these so-called “M cells” within the FAE have unique morphological features, including reduced glycocalyx and microvilli and an intraepithelial pocket beneath the basolateral membrane which contains various populations of lymphocytes and mononuclear phagocytes. In murine PP approximately 10% of the epithelial cells within the FAE are M cells, with the remaining population comprised of absorptive enterocytes and occasional goblet cells (2).

In birds, FAE overlying the follicles of the bursa of Fabricius has been the most studied (3–6). The bursa is a blind-ended sack, connected to the proctodeum by a slit-like duct. The bursa is both a primary lymphoid organ, essential for B cell development and generation of antibody diversity, and a secondary lymphoid tissue (7). Environmental antigenic material gains access to the bursal lumen from the gut or directly from the external environment via the phenomenon known as cloacal drinking, which is the reflexive intake of fluids applied to the external cloacal lips through the cloaca to the proctodeum and bursa (8, 9) The bursal FAE has been proposed to be comprised of a uniform population of antigen sampling epithelial cells (3, 6), interspersed with cells of haematopoietic origin, including macrophages (5). Due to their anatomical location, morphology and apparent role in sampling and transporting luminal antigens, bursal FAE cells have also been called M cells (6, 10, 11). However, their exact relationship to mammalian M cells has not been determined. To date no specific marker(s) for avian antigen sampling FAE cells has been identified, significantly hampering studies of their development and function.

Here we show that the colony-stimulating factor 1 receptor (CSF1R) is expressed in the FAE cells in the avian GALT. In mammals and birds, the proliferation and differentiation of cells of the macrophage lineage is controlled by signals from the CSF1R initiated by two ligands, macrophage colony-stimulating factor (CSF1, also known as M-CSF) and interleukin 34 (IL-34). In mammals, *CSF1R* is expressed in placental trophoblasts throughout embryonic development (12), driven by a cell-type-specific promoter (13). In the embryo itself and in all adult tissues, the expression of *Csf1r* mRNA and protein is restricted to myeloid lineage cells [reviewed in (14)]. In mammals, CSF1R is not expressed in M cells, although CSF1R-dependent macrophages do contribute to the control of M cell differentiation (15). Based upon the findings in mammalian systems, we utilized regulatory elements of the chicken *CSF1R* locus to generate macrophage-restricted reporter transgenic bird lines (16). Transgene expression provided a marker to study the development of the mononuclear phagocyte system of the chick embryo. As in mammals, the transgene was also expressed in heterophils, the avian equivalent of neutrophils (16–18).

We reported previously the surprising finding that *CSF1R*-reporter transgenes are expressed outside the myeloid lineage in FAE-like structures in the respiratory tract (19). Here we show that expression of the reporter genes accurately reflects high expression of CSF1R protein in the FAE of the bursa. Utilizing the *CSF1R*-transgenic chickens we show that the bursal FAE is comprised of different cell types including *CSF1R*-transgene expressing epithelial cells and a phosphatidylserine receptor T cell immunoglobulin and mucin domain-containing 4 (TIM4) expressing phagocyte population which does not express the *CSF1R*-transgene. The expression of our *CSF1R*-transgene allows visualization and functional characterization of distinct cell populations within FAE. Bursal FAE cells that expressed the *CSF1R*-transgene were enriched for transcytotic capacity, exhibiting high levels of apical F-actin and differential staining with the lectins jacalin, PHA-L and SNA compared to other FAE cell populations. Our findings support the view that while the chicken FAE is functionally similar to murine FAE, it is composed of distinct cell types and the bursal FAE antigen sampling cells are likely to have different ontological origins to mammalian M cells.

## Methods

### Chickens and Ethics Statement

*CSF1R*-eGFP/mApple reporter transgenic chickens and wild type control chickens were supplied at 4–6 weeks of age by the National Avian Research Facility, The Roslin Institute, Edinburgh (UK). The production of the transgenic chicken lines used in this study has been previously described (16). Briefly, the chicken *CSF1R* promoter region and Fms intronic regulatory element were used to drive expression of a fluorescent protein (eGFP or mApple) reporter. The birds that carry a copy of this transgene show high levels of expression of the *CSF1R*-reporter in the macrophage, monocyte and dendritic cells lineages and at a much lower level in granulocytes (16). Expression of the *CSF1R*-transgene has not been observed in non-myeloid cell populations, with the exception of the antigen sampling epithelial cells described in this study. The chickens were housed in groups of 22–60 birds and received food and water *ad libitum*. The chickens were maintained under conventional conditions and received standard vaccination scheme against Marek's disease virus, *Eimeria* spp., infectious bronchitis virus, infectious bursal disease virus and Newcastle disease virus. Animals were housed in premises licensed under a UK Home Office Establishment License in full compliance with the requirements of the Animals (Scientific Procedures) Act 1986. Breeding of transgenic chickens was carried out under the authority of Project License PPL 70/8940 with the consent of The Roslin Institute Animal Welfare and Ethical Review Board. Administration of *Salmonella* was undertaken under the authority of Home Office project license PCD70CB48, with the consent of the Ethical Review Committee of the Moredun Research Institute. Chickens inoculated with *Salmonella* were not vaccinated and were housed separately from other birds.

### *In vivo* Treatments

Chickens were administered intracloacally with 100 μl FluoSpheres^®^ Carboxylate-Modified Microspheres (Thermo Fisher Scientific (Life Technologies Ltd.), Renfrew, UK), 2% solids. Bead diameter routinely used was 0.1 μm unless otherwise stated. Birds were culled by cervical dislocation 3 h after administration of beads. Bursae were removed from birds, opened to expose the mucosal surface and were rinsed by dipping 3x in PBS to remove beads that had not been taken up by the FAE. Whole mount imaging is described below. For estimating bead uptake, the rinsed bursae were fixed overnight in 4% paraformaldehyde in phosphate-buffered saline (PBS), washed in PBS and perfused in 30% sucrose in PBS. Selected samples were cryo-embedded in Tissue-Tek^®^ O.C.T.™ Compound (optimal cutting temperature, OCT; Sakura Finetek Europe, Alphen aan den Rijn, Netherlands) and sectioned at 10 μm onto Superfrost Plus (Menzel-Gläser, Braunschweig, Germany) slides.

### Image Analysis

Bead fluorescence was estimated by first defining the *CSF1R*-eGFP FAE as a region of interest (ROI) and calculating the mean fluorescence intensity attributable to beads using ImageJ software (http://rsb.info.nih.gov/ij/; National Institute of Mental Health, Bethesda, Maryland, USA) as described on coded sections (20). Background intensity thresholds were first applied using an ImageJ macro which measures pixel intensity across regions free of beads. The obtained pixel intensity threshold value was then applied in all subsequent analyses. Next, the number of red pixels were automatically counted. For these analyses, data are presented as the proportion of positively-stained red pixels in the specific area of interest (e.g., FAE). In each instance, 10 images were analyzed per chicken, from bursal tissues from multiple chickens per group (*n* = 3 chickens/group).

### Immunohistochemistry

Tissue samples were fixed overnight in 4% paraformaldehyde in PBS, washed in PBS and perfused overnight in 30% sucrose in PBS. Selected samples were cryo-embedded in OCT and sectioned at 10 μm onto Superfrost Plus slides. For anti-CSF1R staining, tissues were embedded in OCT without fixation, sectioned and fixed with 100% methanol at 4°C for 10 min before air-drying for 1 h at room temperature. All primary antibodies and lectins used in this study are shown in Tables 1, 2, respectively. Alexa Fluor^®^ 647 phalloidin (Thermo Fisher Scientific (Life Technologies Ltd.), Renfrew, UK, diluted 1/50) was used for F-actin staining. All slides were blocked for 1 h in 2.5% skimmed milk powder (Oxoid Ltd., Basingstoke, UK), 2.5% normal horse serum (Sigma, Gillingham, UK), 0.1% Triton X-100 (Sigma, Gillingham, UK) in PBS (MST-PBS). Isotype matched antibody controls (Table 1) were added at the same concentration as primary antibodies. Control staining for lectin was performed by excluding labeled lectin. Antibodies were diluted in MST-PBS and all washes were in PBS. Primary antibodies (Table 1), including rabbit anti-GFP Alexa Fluor 488, were incubated at 4°C overnight, followed by incubation with secondary antibodies for 2 h (donkey anti-rabbit IgG Alexa Fluor 488, donkey anti-goat IgG Alexa Fluor 488, donkey anti-mouse IgG Alexa Fluor 594/647; all used at 1/300 dilution; Thermo Fisher Scientific (Life Technologies Ltd.), Renfrew, UK), and mounted in ProLong^®^ Gold Antifade Mountant (Thermo Fisher Scientific (Life Technologies Ltd.), Renfrew, UK). Where appropriate, sections were counterstained with 1 μg/ml 4′, 6′-diamidino-2-phenylindole (DAPI; Sigma, Gillingham, UK) in the final incubation step. Samples were imaged using an inverted confocal microscope (Zeiss LSM710).

**Table 1:** Primary antibodies used in this study.

**Primary antibody (clone; catalog number)**	**Host**	**Target**	**Dilution**	**References; source**
LAMP1 (LEP100 IgG (formerly called cv24); LEP100 IgG)	Mouse IgG1	Late endosomes/lysosomes	1/50	Lippincott-Schwartz and Fambrough (21); DHSB^*^
CSF1R (ROS-AV170; MCA5956GA)	Mouse IgG1	Macrophage colony-stimulating factor 1 receptor	1/100	Garcia-Morales et al. (22); Bio-Rad, Dalkeith UK).
CD45 (AV53; N.A.)	Mouse IgG1	All chicken hematopoietic cells, except erythrocytes and plasma cells,	1/1,000	Institute for Animal Health, UK
Annexin A10 (N.A.; HPA005469)	Rabbit IgG (polyclonal)	Annexin A10	1/200	Atlas antibodies
CD44 (AV6; N.A.)	Mouse IgG1	B-cells, T-cells, monocytes and some epithelial cells	1/500	Institute for Animal Health, UK
L-plastin (N.A.; D-16)	Goat IgG (polyclonal)	Haematopoietic cells	1/200	Santa Cruz Biotechnology, Inc., Germany
EEA1 (N.A.; ab2900)	Rabbit IgG (polyclonal)	Early endosomes	1/200	Abcam, UK
Lysozyme (N.A.; ab391)	Rabbit IgG (polyclonal)	Lysozyme (hen egg white)	1/200	Abcam, UK
Gamma-actin (2A3; MCA5776GA)	Mouse IgG2b	Cytoskeleton	1/200	Dugina et al. (23); Bio-Rad, Dalkeith, UK
TIM4 (JH9)	Mouse IgG1	Phosphatidylserine receptor	1/1,000	Hu et al. (24); N.A.
IgG1 isotype control, mouse (GR13.1)	Mouse IgG1	anti-ovine CD335	Various	Bio-Rad, Dalkeith UK
IgG2b isotype control	Mouse IgG2b	Anti-rat surface protein	Various	Bio-Rad, Dalkeith UK
Rabbit control IgG	Rabbit IgG	Purified rabbit IgG	Various	Bio-Rad, Dalkeith UK

* *Developmental Studies Hybridoma Bank, Iowa City, IA*.

**Table 2:** Lectins used in this study.

**Lectins (catalog number)**	**Target**	**Dilution**	**Manufacturer**
Phaseolus vulgaris- Leucoagglutinin (PHA-L—FITC; FL-1111)	Complex oligo-saccharides Containing galactose, N-acetyl-glucosamine, mannose	1:200	Vector Laboratories, UK
Jacalin—biotin (B-1155)	O-glycoproteins	1:500	Vector Laboratories, UK
Wisteria Floribunda Lectin (WFA—FITC; FL-1351)	N-acetylgalactosamine linked α or β to the 3 or 6 position of galactose	1:200	Vector Laboratories, UK
Sambucus Nigra (SNA—FITC; FL-1301)	Sialic acid attached to terminal galactose in α-2,6 and to a lesser degree, α-2,3	1:200	Vector Laboratories, UK

For whole mount immunostaining of the bursal FAE, 0.5 × 0.5 cm sections of 4% PFA fixed bursa tissue were placed in a 1.5 ml Eppendorf tube and blocked with 1 ml of 2.5% normal horse serum, 0.1% Triton X-100 in PBS for 1 h at room temperature. Primary antibodies (Table 1) and lectins (Table 2) were added to fresh blocking solution at the indicated dilutions and samples incubated with rocking at 4°C overnight. Isotype matched antibody controls (Table 1) were added at the same concentration as primary antibodies. Control staining for lectin was performed by excluding labeled lectin. Tissue samples were washed for 30 min in PBS and either secondary antibodies, fluorochrome labeled streptavidin or fluorochrome labeled Phalloidin (Thermo Fisher Scientific (Life Technologies Ltd), Renfrew, UK) were added to the samples in fresh blocking solution, then incubated at room temperature with rocking for 2 h. Tissue samples were washed for 30 min in PBS before imaging.

### Microscopy

Images of cryosections were captured using Zeiss ZEN software (Carl Zeiss Ltd., Cambridge, UK) and analyzed using Imaris software, version 8.2 (Bitplane, Zürich, Switzerland). For imaging of whole mount immunostained tissues, a 35 × 10 mm petri dish was modified by cutting a hole in the lower section and fixing a coverslip over the hole with nail polish. Stained bursas were placed on the coverslip, with the epithelial surface in contact with the cover slip. Samples were imaged using an inverted confocal microscope (Zeiss LSM710). For 3D-rendering, confocal z-stacks were created by obtaining images at 0.45 μm intervals. Images were captured using Zeiss ZEN software and analyzed using Imaris software, version 8.2.

### Bacterial Strains and Culture Conditions

A defined mutant of ST4/74 *nal*^*R*^ lacking *prgH* was generated by λRed recombinase-mediated integration of linear PCR products as previously described (25). Briefly, the pKD4-encoded kanamycin (kan) resistance cassette was amplified with primers (*prgH*::*kan*F 5′-gtgcggtaatctgctgcttatcgagaacgacagacatcgctaacagtatatgtgtaggctggagctgcttc-3′and *prgH*::*kan*R 5′-agatagcctgaccaaggtgttgccataatgacttccttatttacgttaaaccatatgaatatcctcctta-3′) which include 51bp extensions homologous to the flanking regions of *prgH*. This amplicon was electroporated into ST4/74 expressing λRed from plasmid pKD46 recombinants selected as described (26) and confirmed by PCR with primers flanking *prgH* (*prgH*::*kan*_diag F 5′-ggggatgatggtttcttttaa-3′ and *prgH*::*kan*_diag R 5′-ccgagagcttactctgatac-3′). The mutant was further validated by Western blotting of secreted proteins for the T3SS-1 effector SipC (not shown). Both ST4/74 *nal*^*R*^ and its derivative ST4/74 *nal*^*R*^ Δ*prgH*::*kan* were transformed with plasmid pFC(c)G(i) which constitutively expresses mCherry (27), and expression of mCherry confirmed by fluorescence microscopy.

### Infection of *CSF1R*-eGFP Chickens With mCherry-expressing *Salmonella* Strains

Thirty-six *CSF1R*-eGFP reporter transgenic chickens were housed under specified pathogen-free conditions from the day of hatch and a sterile irradiated diet (DBM Ltd., UK) and water were provided *ad libitum*. At 4 weeks of age, 18 birds were inoculated intracloacally with 100 μl of ST4/74 *nal*^*R*^ (3.35 × 10^8^ CFU) or ST4/74 *nal*^*R*^ Δ*prgH::kan* (3.35 × 10^8^ CFU) expressing mCherry from plasmid pFC(c)G(i). Birds inoculated with the two strains were housed separately and 6 birds from each group were culled by cervical dislocation at 30 min, 1 and 3 h post-infection. The bursa was collected and split into 2 parts—one for bacterial enumeration and one for immunofluorescence microscopy. To enumerate bacteria, 1 g of bursa was added to 9 ml of PBS and dissociated to a single-cell suspension using a gentleMACS Dissociator in gentleMACS C Tubes (Miltenyi Biotech, Woking, UK) using the Intestine programme. Ten-fold serial dilutions of the resulting single cell suspension were plated onto MacConkey agar supplemented with 20 μg/ml of nalidixic acid and 100 μg/ml ampicillin and incubated overnight at 37°C to recover total bacteria. An aliquot of the single cell suspension was also treated with gentamicin at a final concentration of 100 μg/ml and incubated at 37°C for 30 min to kill extracellular bacteria. Ten-fold serial dilutions of the gentamicin-treated suspension were plated onto MacConkey agar supplemented with 20 μg/ml of nalidixic acid and 100 μg/ml ampicillin and incubated overnight at 37°C to enumerate intracellular bacteria.

### Statistical Analysis

Unless indicated otherwise, differences between groups were analyzed by a Student's *t*-test. The significance of differences in total and intracellular bacterial numbers detected in the bursa of birds inoculated with the wild-type ST4/74 *nal*^*R*^ or its *prgH* mutant, ST4/74 *nal*^*R*^ Δ*prgH::kan*, were determined using *post-hoc* Dunnet's tests following fitting of a third order hierarchical general linear model, taking into account interactions between the time of sampling, treatment group and localization of bacteria using the Minitab 17 software (Minitab, Coventry, UK). In instances where there was evidence of non-normality (identified by the Kolmogorov-Smirnov, D'Agostino & Pearson omnibus, or Shapiro-Wilk normality tests), data were analyzed by a Mann-Whitney *U*-test. Values of *P* < 0.05 were accepted as significant. All data are presented as mean ± SD.

## Results

### *CSF1R*-Transgene Expression Allows Differential Visualization of Mononuclear Phagocytes and Epithelial Cells That Comprise the FAE of the Bursa of Fabricius

**Figure 1:**
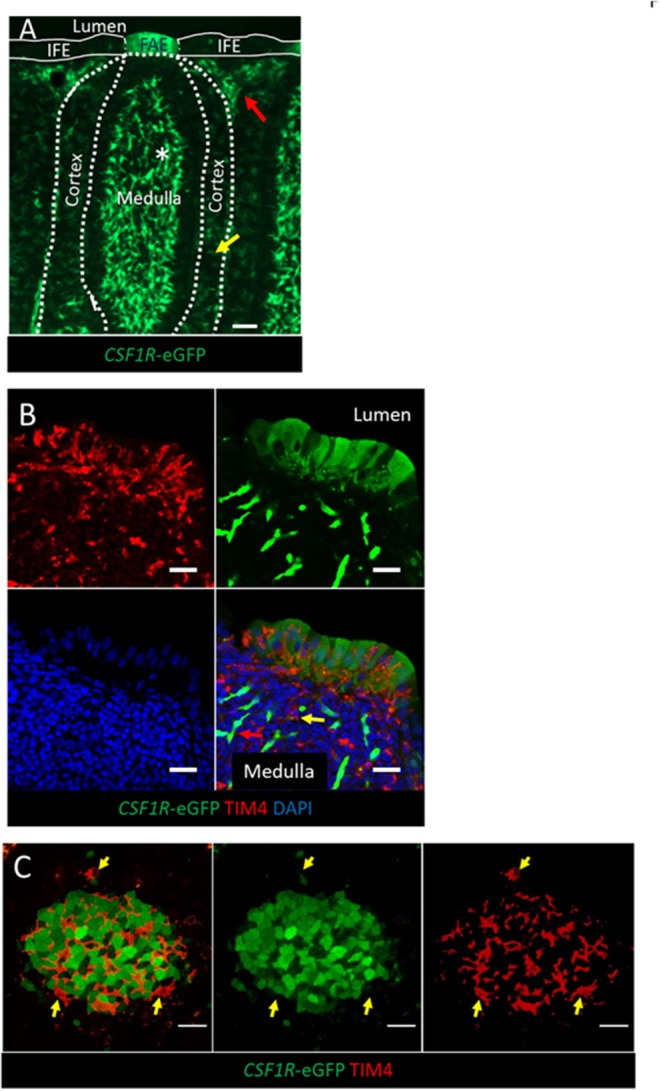
The expression of the *CSF1R*-transgene in the bursal FAE. **(A)** *CSF1R*-eGFP expressing cells are found in the bursal medulla, cortex, inter-follicular mesoderm and FAE. *CSF1R*-eGFP expressing; bursal secretory dendritic cells (white asterix) are found in the medulla, whereas uncharacterized subsets of *CSF1R*-eGFP expressing phagocytes are found in the cortex (yellow arrow) and inter-follicular regions (red arrow); scale bar = 50 μm. **(B)** The bursal FAE is composed of *CSF1R*-transgene expressing epithelial cells and TIM4+ phagocytes (red). A representative *CSF1R*-eGFP expressing bursal secretory dendritic cell and TIM4+ *CSF1R*-eGFP- phagocyte in the medulla are marked with a red and yellow arrow, respectively; scale bar = 10 μm. **(C)** Confocal microscopy section of a single bursa FAE, TIM4+ phagocytes (red) do not express *CSF1R*-eGFP (yellow arrows), unlike the majority of FAE epithelial cells (green cells); scale bar = 20 μm. Representative of three independent experiments.

**Figure 2:**
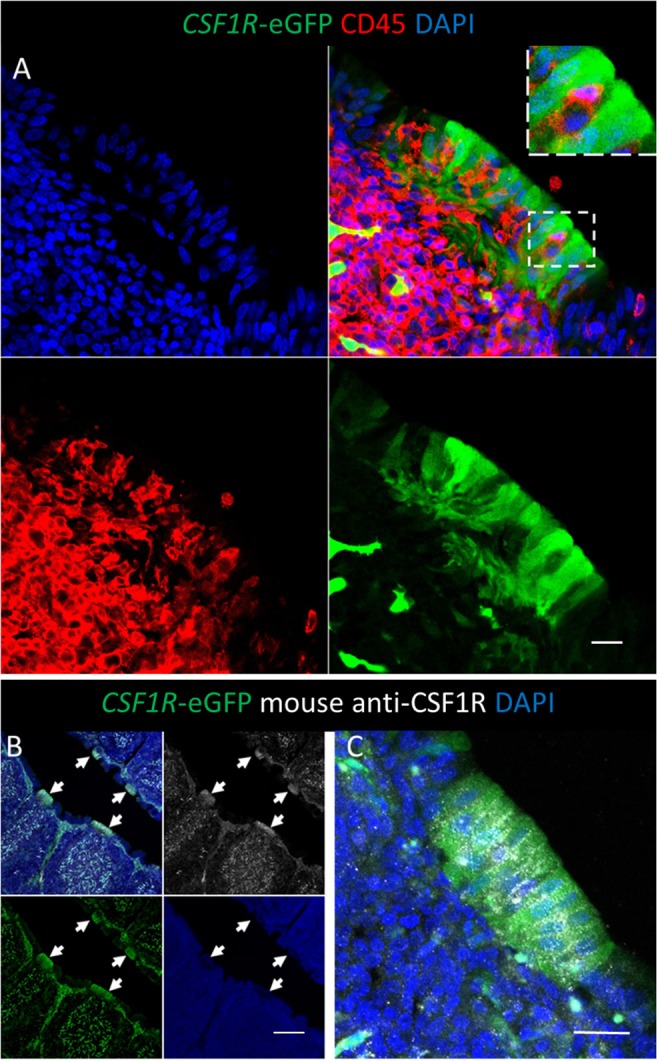
Bursal FAE cells express the *CSF1R*-eGFP transgene and CSF1R protein. **(A)** The bursal FAE is a heterogeneous structure consisting of *CSF1R*-transgene expressing epithelial cells (green) and CD45+ haematopoietic cells (red). The insert shows the detail of a CD45+ cell located between two epithelial cells; scale bar = 10 μm. **(B)** CSF1R protein is expressed in the same cells as the *CSF1R*-eGFP transgene in the bursa of Fabricius, including the FAE (**B**; white arrows); scale bar = 100 μm. **(B,C)** CSF1R protein is concentrated on the basolateral membrane of CSF1R-eGFP+ FAE epithelial cells and distributed in a punctate manner; scale bar = 20 μm. Representative of three independent experiments.

**Figure 3:**
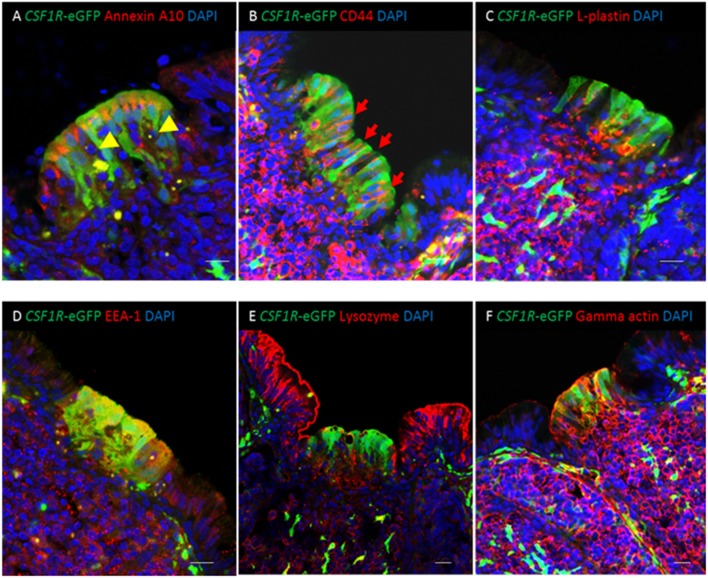
Co-expression of putative M cell markers in the bursal FAE. **(A)** Cytoplasmic expression of the putative M cell marker annexin A10 is seen in *CSF1R*-eGFP FAE cells. *CSF1R*-eGFP negative cells containing condensed and fragmented cell nuclei (yellow arrowheads) are abundant in the FAE. **(B)** Membrane associated staining for CD44 (red) is seen in *CSF1R*-eGFP epithelial cells (**B**; red arrows); **(C)** L-plastin+ cells (red) are found in the medulla and FAE. **(D)** *CSF1R*-eGFP FAE are enriched for EEA-1 staining compared with IFE cells, whereas **(E)** IFE cells are enriched for lysozyme compared to *CSF1R*-eGFP expressing FAE cells. **(F)** Gamma actin (red) is expressed in the bursal *CSF1R*-eGFP expressing FAE cells, but not the IFE cells. Scale bar for all images = 10 μm. Representative of 7–15 individual follicles, 2–5 experiments.

Consistent with expression of CSF1R protein, and macrophage-specific transcription, the *CSF1R*-eGFP transgene labels cells with morphology typical of mononuclear phagocytic cells in the medulla, cortex and inter-follicular regions of the bursal follicle (Figures 1A,B). *CSF1R*-transgene expressing cells in the medulla are bursal secretory dendritic cells (16), whereas *CSF1R*-transgene expressing cells in the cortex and inter-follicular regions have yet to be defined. The bursal FAE also showed strong expression of the *CSF1R*-eGFP transgene (Figure 1A). While most of the studies described here use the *CSF1R*-eGFP reporter transgene (to facilitate double labeling), expression of the independent *CSF1R*-mApple reporter transgene was also observed in the same subsets of cells (see below). While the bursal follicle, including the FAE, contains macrophages, these macrophages do not express typical avian macrophage markers (28, 29). In common with other macrophages within bursal follicles, as opposed to inter-follicular populations (30), we found that macrophages in the FAE did not express the *CSF1R*-transgene but could be identified by the expression of TIM4 (Figures 1B,C). Differential *CSF1R*-transgene expression and TIM4 staining of cells in the bursal FAE indicated that *CSF1R*-transgene expression in the FAE was not due to the presence of macrophages (Figures 1B,C). Indeed, *CSF1R*-transgene expressing cells were also negative for the pan-haematopoietic cell marker CD45 (Figure 2A). The intensity of eGFP expression between individual cells of the FAE was somewhat variable, suggesting dynamic expression of the *CSF1R*-transgene (Supplementary Figure 1D). The morphology of bursal *CSF1R*-transgene expressing cells of the FAE was consistent with their identification as antigen sampling bursal FAE cells, having a tapered or branched basal cytoplasm [Figure 2A; (3, 31–34)]. Murine M cells have a basolateral pocket structure, which contains lymphocytes and mononuclear phagocytes (2). Basolateral pockets were not observed in *CSF1R*-transgene expressing cells of the bursal FAE, although TIM4+ macrophages were observed intimately associated with the basolateral surface and between individual *CSF1R*-transgene expressing cells of the FAE (Figure 1B). To confirm the association of the *CSF1R*-transgene with expression of CSF1R protein we used an anti-chicken CSF1R antibody described previously (22). The FAE cells clearly expressed both the *CSF1R*-transgene and abundant CSF1R protein (Figures 2B,C; Supplementary Figure 1). CSF1R protein was detected in the FAE of both wild type and transgenic birds (Figure 3; Supplementary Figure 1B), whereas *CSF1R*-transgene expression was only detected in transgenic birds (Supplementary Figures 1A,C). CSF1R protein staining was punctate and restricted to the basolateral membrane (Figures 2B,C) suggesting that it may function as a receptor for one of the two ligands, CSF1 and IL-34, acting directly on the FAE.

### *CSF1R*-transgene Expressing Bursal FAE Cells Stain With Putative M Cell Markers

We examined the distribution of markers previously reported as putative pan-M cell markers (annexin A10, CD44, L-plastin), on the basis of enriched gene expression in chicken bursal FAE cells and murine PP M cells (6), on *CSF1R*-transgene expressing FAE cells. Expression of annexin A10, CD44, and L-plastin was enriched in the FAE in comparison to the inter-follicular epithelium (IFE) (Figures 3A–C). Annexin A10 staining was largely restricted to the apical cytoplasm of FAE cells (Figure 3A, Supplementary Figure 2A) while CD44 expression was observed on the basolateral membranes of FAE cells and the majority of lymphocytes in the medulla and cortex (Figure 3B). L-plastin staining differentiated the FAE from the IFE (Figure 3C), but this staining was not specifically associated with *CSF1R*-transgene expressing FAE cells (Figure 3C). Of these three putative M cell markers, annexin A10 appeared to be the most specific for *CSF1R*-transgene expressing bursal FAE cells, although some scattered cell staining was observed throughout the bursal follicle (Supplementary Figure 2A). Previously we have shown that the *CSF1R*-transgene is also expressed in FAE cells of bronchus-associated lymphoid tissue [BALT; (19)]. In contrast to bursal *CSF1R*-eGFP expressing FAE cells, in the lung we observed that annexin A10 expression was not restricted to *CSF1R*-eGFP expressing FAE cells indicating that this is not a universal marker of *CSF1R*-eGFP expressing FAE cells in chicken MALT structures (Supplementary Figure 2B). Bursal FAE and IFE cells could also be distinguished from each other on the basis of differential staining for lysozyme, early endosome antigen 1 (EEA-1) and gamma-actin (Figures 3D–F). Staining for CD44, L-plastin, EEA-1 and gamma-actin was continuous between the FAE and the medulla indicating that these markers are not specific for FAE *CSF1R*-transgene expressing cells and raised the possibility that positive staining for all these markers in the FAE was due to the presence of haematopoietic cells. Analysis of other GALT structures confirmed that the *CSF1R*-transgene was also expressed in FAE cells in the caecal tonsil and proctodeum (Supplementary Figure 3), demonstrating that *CSF1R*-transgene expression is a marker for FAE cells in diverse chicken MALT.

### F-actin and Jacalin Lectin Are Reliable Markers for Antigen Sampling Cells That Express *CSF1R*-transgene in the FAE

**Figure 4:**
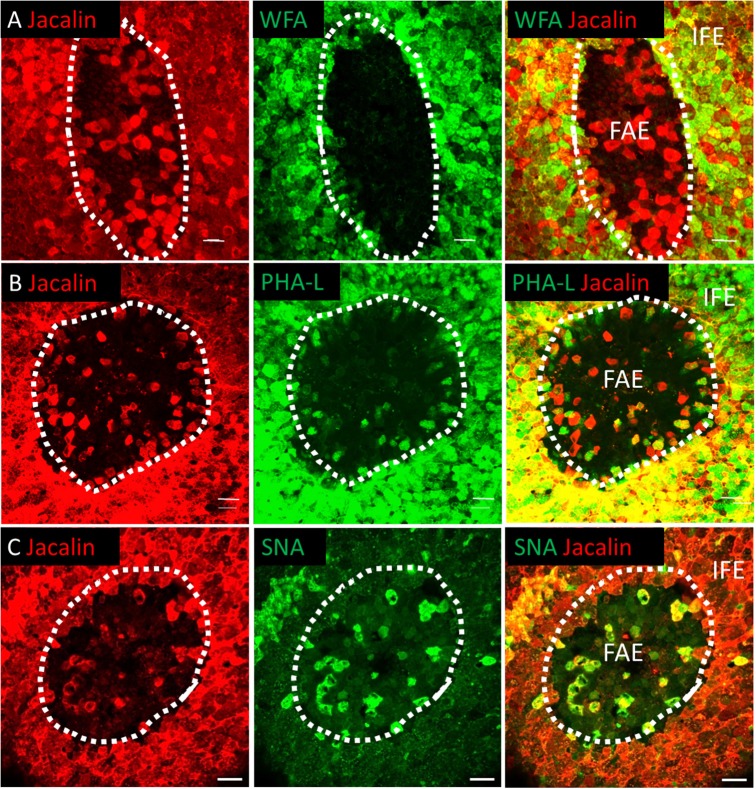
The bursal FAE is a heterogeneous structure. Double label lectin staining was performed on bursal whole mount preparations. Dotted line denotes the extent of the bursal FAE. Confocal analysis indicates that Jacalin+ cells (red) are found in the bursa FAE and can be differentiated from IFE mucin producing cells due to the lack of WFA lectin co-staining **(A)**. Jacalin+ FAE cells also co-stain with PHA-L **(B)** and SNA lectins **(C)**. Scale bars = 10 μm Representative of 5–15 individual follicles.

Previously bursal FAE and IFE cells, the latter largely consisting of mucin-secreting cells (5, 31, 35), have been distinguished from each other on the basis of lectin binding on tissue sections (11, 36, 37). *CSF1R*-transgene expression distinguished the bursal FAE and IFE but some FAE cells appeared to lack *CSF1R*-transgene expression (Supplementary Figure 1D). Whole-mount lectin staining was carried out to further characterize the bursal FAE heterogeneity (Figure 4). None of the lectins tested stained all FAE cells, but several lectins stained a sub-population (Figures 4A–C). WFA stained IFE cells but was largely excluded from the FAE (Figure 4A). Jacalin and PHA-L lectins labeled all IFE cells, as well as scattered cells within the FAE (Figure 4B). Most of the jacalin^+^ FAE cells were SNA^+^ (Figure 4C). The lack of WFA and presence of SNA staining suggested that the jacalin/PHA-L/SNA^+^ cells in the FAE were not mucin-producing IFE cells.

**Figure 5:**
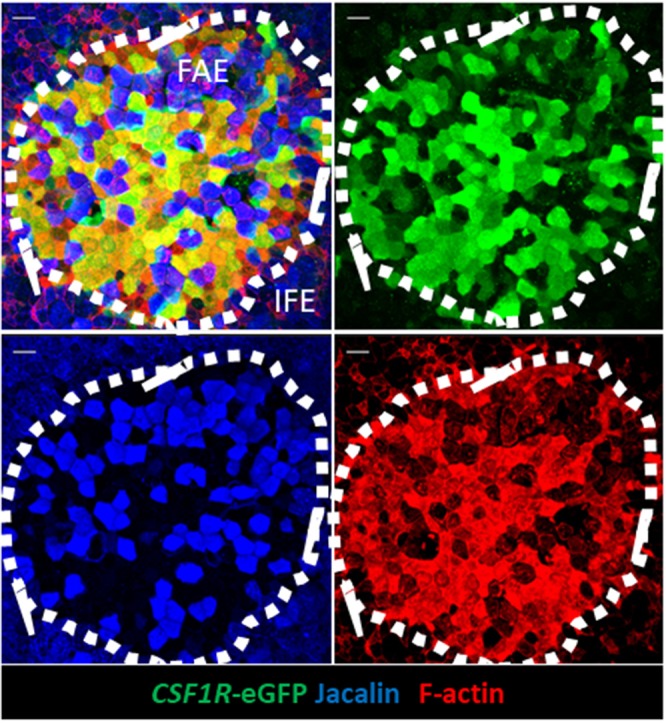
*CSF1R*-transgene expressing, but not jacalin lectin staining, bursa FAE cells express high levels of F-actin. Triple label staining was performed on bursal whole mount preparations. The mucosal surface of the bursa of Fabricius is shown rendered from the Z-stack dataset of 57 × 0.73 μm images using Imaris software. F-actin staining is concentrated in the FAE. Jacalin+ FAE cells have much reduced F-actin staining compared to *CSF1R*-transgene expressing FAE cells. Representative of 10 individual follicles. Scale bar = 10 μm.

Endocytosis and transcytosis involve remodeling of membrane and the cytoskeleton lying beneath the cell membrane (38). Murine M cells have high levels of F-actin and actin-associated proteins associated with the apical cell membrane (38). To determine if this was the case for *CSF1R*-transgene expressing FAE cells we investigated F-actin expression in bursal IFE and FAE cells by whole-mount IHC (Figure 5). F-actin, visualized by phalloidin staining, was higher in the bursal FAE, compared to the IFE (Figure 5; Supplementary Figure 4). Within the FAE, *CSF1R*-transgene positive FAE cells expressed high levels of F-actin (Figure 5), whereas jacalin^+^ FAE cells had visibly lower levels of F-actin (Figure 5). F-actin was concentrated in the apical surface of *CSF1R*-transgene positive FAE cells (Supplementary Movie 1). We conclude that the FAE contains two distinct populations of epithelial cells distinguished by CSF1R expression, lectin binding and apical F-actin. Staining patterns of lectins and F-actin on bursa of Fabricius epithelial cells is summarized in Table 3.

**Table 3:** Summary of F-actin and lectin staining on the bursa of Fabricius.

	**IFE**	**FAE**	
		***CSF1R*-Tg^**+ve**^**	***CSF1R*-Tg^**-ve**^**
F-actin	+	+++	+
Jacalin	+++	–	+++
WFA^*^	+++	–	–
PHA-L^*^	+++	–	++
SNA^*^	–/+	–	+++

* *Staining on FAE transgene (Tg) +ve/–ve cells inferred by co-staining with jacalin*.

### *CSF1R*-transgene Expression Identifies Antigen Sampling FAE Cells in the Chicken Bursa of Fabricius

**Figure 6:**
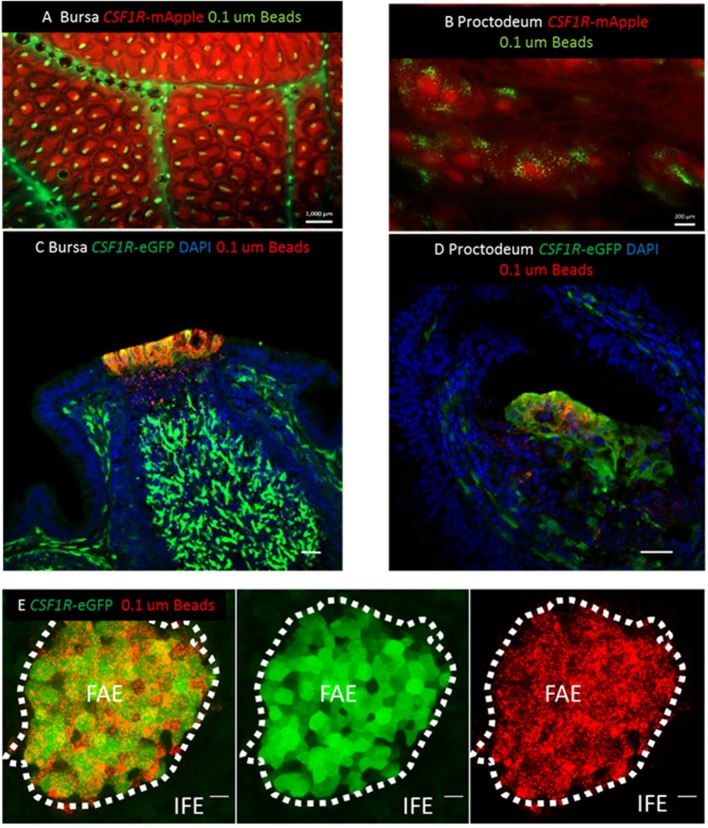
*CSF1R*-transgene expression identifies functional antigen sampling FAE cells in the avian bursa of Fabricius. **(A)** Three hours post-intracloacal challenge 0.1 μm fluorescent latex beads can be seen associated with individual lymphoid follicles of the *CSF1R*-mApple expressing bursa (scale bar = 1000 μm) and **(B)** proctodeum (scale bar = 200 μm) in mucosal whole mount views. **(C)** Three h post-intraclocal challenge beads are found in *CSF1R*-eGFP expressing cells in the bursal FAE and tissue underlying the FAE, indicative of active transcytosis by *CSF1R*-eGFP expressing FAE epithelial cells (scale bar = 50 μm). **(D)** *CSF1R*-eGFP expressing proctodeum FAE cells also take up beads 3 h post-intraclocal challenge (scale bar = 50 μm). **(E)** Mucosal surface of the bursa of Fabricius is shown rendered from the Z-stack dataset of 53 × 0.73 μm images using Imaris software. Uptake of 0.1 μm beads (red) is tightly associated with *CSR1R*-eGFP expressing bursal FAE cells, but not *CSF1R*-eGFP negative IFE cells; scale bar = 20 μm. Representative of 3 independent experiments.

The apical F-actin staining in *CSF1R*-transgene expressing cells in the bursal FAE suggests that these cells have endocytic and transcytotic capacity and may be functional equivalents of mammalian M cells. To test this hypothesis, chickens were inoculated with fluorescent 0.1 μm latex beads via the intracloacal route. Three hours after intracloacal inoculation, beads were localized to discrete regions of the mucosal surface in both the bursa and proctodeum (Figures 6A–D). To improve image quality at low magnification, whole mount imaging of live tissues *CSF1R*-mApple transgenic chickens was used in this experiment (Figures 6A,B), whereas for PFA fixed tissues *CSF1R*-eGFP transgenic chickens were used (Figures 6C,D), as PFA fixation reduces mApple fluorescence intensity. Figure 6E shows whole-mount staining and 3D-reconstruction of an individual bursa FAE, 3 h post-intracloacal challenge with beads. The uptake of beads was confined to the *CSF1R*-eGFP expressing FAE, but not the *CSF1R*-transgene negative IFE. This analysis clearly showed that beads were located specifically in the *CSF1R*-transgene expressing FAE cells in both the bursa of Fabricius (Figures 6A,C,E) and the proctodeum (Figures 6B,D).

**Figure 7:**
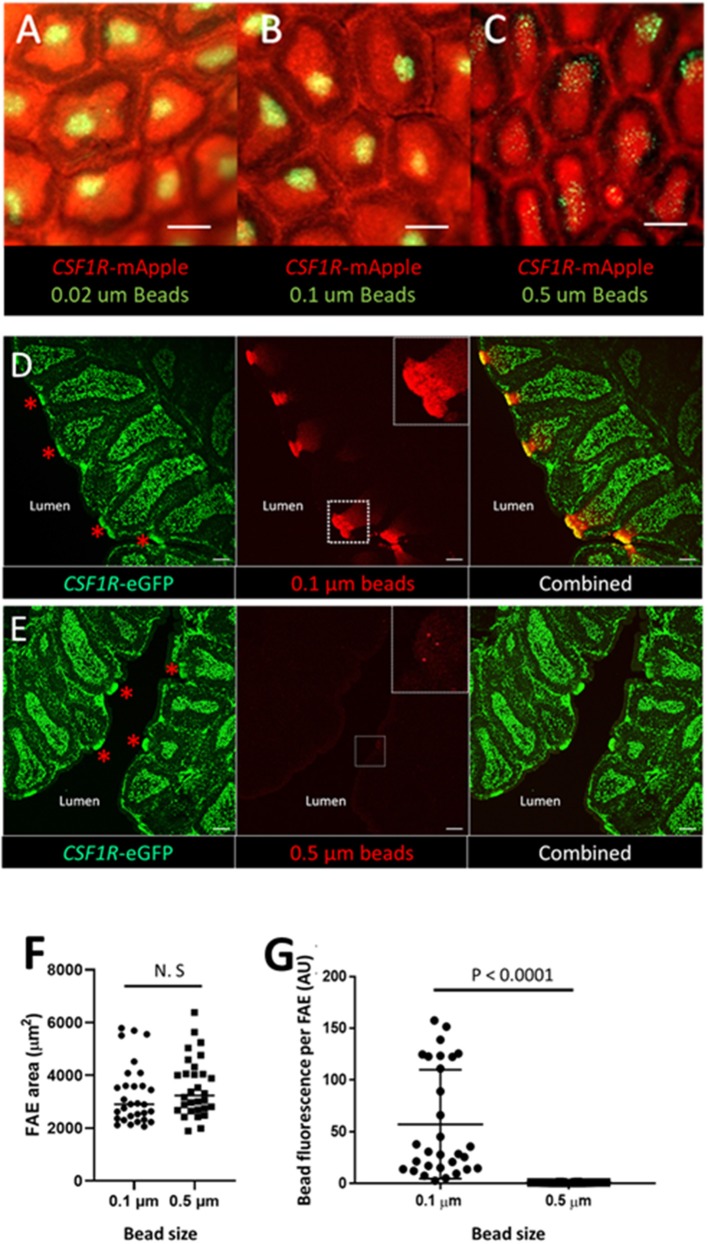
The bursal FAE efficiently takes up latex beads in the 0.02–0.1 μm, but not the 0.5 μm size range. Microscopic view of the mucosal surface of bursa from *CSF1R*-mApple transgenic birds 3 h post intracloacalchallenge. **(A,B)** 0.02 and 0.1 μm beads are efficiently taken up from the bursa lumen 3 h post intraclocal challenge (**A,B**; scale bar = 200 μm), **(C)** in contrast very few 0.5 μm beads are taken up by the bursa FAE (**C**; scale bar = 200 μm). **(D)** Representative confocal image of section of bursa 3 h post intra-cloacal challenge with 0.1 μm beads. Insert shows bead red fluorescence due to bead uptake, which represents many thousands of beads. Red asterix indicate the location of individual FAE. Scale bar = 100 μm. **(E)** Representative confocal image of section of bursa 3 h post intra-cloacal challenge with 0.5 μm beads. Insert shows bead red fluorescence due to bead uptake. In this example, three beads are observed. Red asterix indicate the location of individual FAE. Scale bar = 100 μm. Representative of 5–15 individual experiments. **(F)** Quantification of the area of individual FAE. (*P* = 0.369, *t*-test; data derived from 10 FAE areas/chicken, *n* = 3 chickens/group). N.S. = not significant. **(G)** Mean bead fluorescence per FAE, AU = arbitrary units. Morphometric analysis revealed a significant difference in uptake of 0.1 μm compared to 0.5 μm beads by individual FAE (*P* < 0.0001, Mann-Whitney *U*-test; data derived from 10 FAE areas/chicken, *n* = 3 chickens/group).

The antigen sampling potential of *CSF1R*-transgene expressing FAE cells was further characterized by examining the ability of bursal FAE cells to capture and transport fluorescently labeled latex beads of different sizes and standardized shape. Three hours after intracloacal inoculation, latex beads in the size range of 0.02–0.1 μm were efficiently taken up by the bursal FAE (Figures 7A,B). Uptake of 0.01–0.2 μm beads was uniform, with the beads labeling the entire FAE (Figures 7A,B,D). By contrast, few follicles internalized 0.5 μm sized beads and where this occurred, uptake was non-uniform across the FAE (Figures 7C,E). Three hours post-cloacal challenge 0.1 μm bead uptake by the bursal FAE was significantly higher than for 0.5 μm beads (Figures 7F,G). Indeed, a maximum number of 5 beads in a single FAE was observed for the 0.5 μm bead challenge group, indicating that the bursal FAE does not readily take up particles of this size.

**Figure 8:**
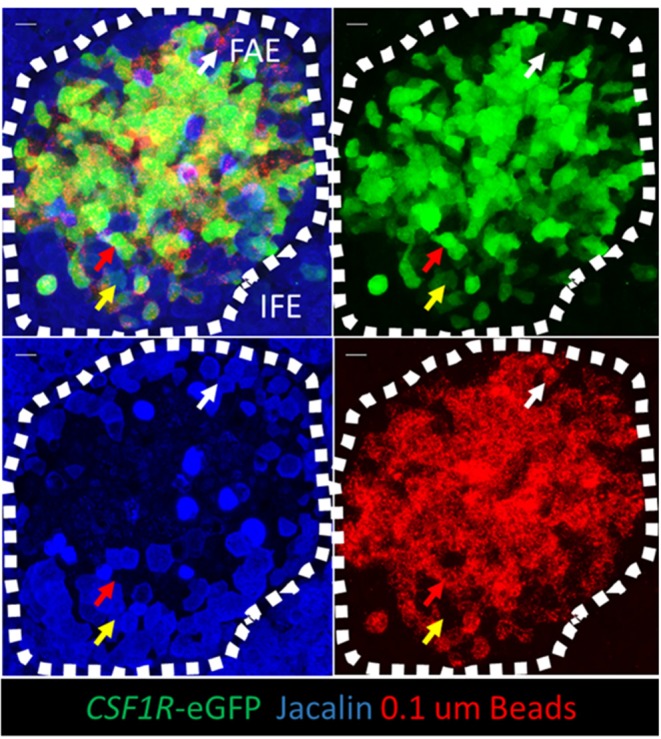
*CSF1R*-transgene expressing bursal FAE cells are the functional equivalent of mammalian M cells. Jacalin lectin staining was performed on whole mount bursal tissue. The mucosal surface of the bursa of Fabricius is shown rendered from the Z-stack dataset of 53 × 0.73 μm images using Imaris software. Three hours post-intraclocal challenge the majority of 0.1 μm latex beads are associated with *CSF1R*-transgene expressing FAE cells (red arrow), but not jacalin lectin staining FAE cells. Occasionally jacalin+ FAE cells expressing low levels of the *CSF1R*-transgene are observed (yellow arrow) and also jacalin+ *CSF1R*-transgene negative FAE cells are found in association with latex beads (while arrow). Scale bar = 10 μm. Representative of 5 individual follicles.

The uptake of 0.1 μm beads by bursal FAE cells was examined in more detail by whole mount immunofluorescence staining and confocal analysis (Figure 8; Supplementary Figure 5). Fifteen minutes after intracloacal inoculation, 0.1 μm latex beads were concentrated immediately below the apical surface of FAE cells (Supplementary Figure 5). Three hours after intracloacal inoculation, 0.1 μm latex beads were concentrated within *CSF1R*-transgene positive FAE cells (Figure 8), located at the basolateral surface of FAE cells (Supplementary Figure 5; Supplementary Movie 2). Occasionally jacalin^+^ FAE cells were observed to weakly express the *CSF1R*-transgene (Figure 8) and in some cases these jacalin^+^ FAE cells were also associated with beads (Figure 8). In contrast, in *CSF1R*-transgene^+^ jacalin^−^ cells the beads were largely associated with the basolateral cell membranes (Supplementary Movie 2). We conclude that *CSF1R*-transgene expressing cells are the subset of FAE that efficiently internalizes smaller microparticles, but they do not internalize particles approaching the size of bacteria.

### *Salmonella* Specifically Enters the Bursal FAE

**Figure 9:**
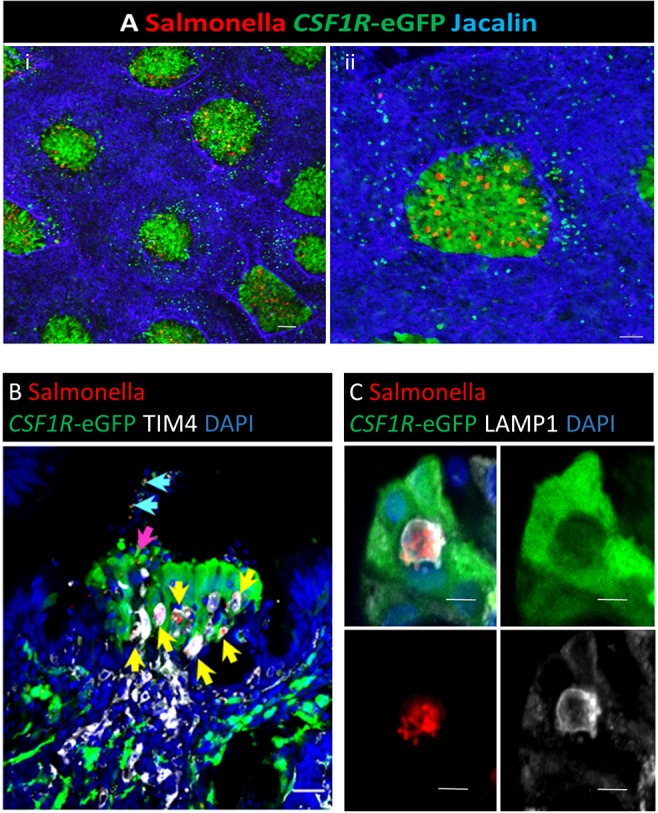
*Salmonella* Typhimurium enters the bursal FAE. Whole mount staining of the bursa of Fabricius. **(A)** Three hours post-intracloacal challenge *Salmonella* (red) are associated with the *CSF1R*-eGFP-transgene expressing FAE cells (green), few bacteria are attached to the IFE regions (highlighted by Jacalin lectin staining, Blue); Scale bar = 1,000 μm. An enlarged image of an individual bursal follicle is shown in (ii); Scale bar = 2,000 μm. **(B)** Confocal analysis of an individual FAE invaded by *Salmonella*. The vast majority are contained within TIM4+ cells (yellow arrow), rarely individual bacteria are observed in association with *CSF1R*-eGFP FAE cells (pink arrow) or cellular debris in the lumen (blue arrows); Scale bar = 10 μm. **(C)** Clusters of bacteria in the FAE are located within LAMP1+ vacuoles; Scale bar = 5 μm. Representative of 20 follicles.

*Salmonella* Typhimurium exhibits tropism toward mammalian M cells as a portal of entry across the gut epithelium (39, 40). Invasion of mammalian M cells is partly dependent on expression of the Type III secretion system-1 (T3SS-1) encoded by *Salmonella* pathogenicity island 1 (SPI-1), which injects a set of bacterial effector proteins that remodel the subcortical actin cytoskeleton to engulf the pathogen (41, 42). To investigate whether *S*. Typhimurium specifically invades bursal *CSF1R*-transgene expressing FAE cells and whether this requires the T3SS-1, we infected 4-week old *CSF1R*-transgenic chickens with mCherry-expressing wild-type *S*. Typhimurium ST4/74 or a Δ*prgH::kan* mutant. PrgH is a key component of the secretion apparatus and null mutants are known to be impaired in intestinal colonization (26), ileal invasion (43) and induction of enteritis (44) in mammals. There was no significant difference in total counts between the wild-type and the Δ*prgH::kan* strains (*P* = 0.33), demonstrating that within this short period of time the net survival and replication of the Δ*prgH::kan* mutant within the bursa was not impaired. We detected significant differences in total counts compared to gentamicin-protected bacteria for both the wild-type and the Δ*prgH::kan* strains at all time-points (*P* < 0.001 for all comparisons), indicating that only a small fraction of the total bacteria present were intracellular. When taking into account all three time intervals, there was significant difference in intracellular counts between the wild-type and the Δ*prgH::kan* strain (*P* < 0.001; c. 1 log_10_ CFU/g at each time), indicating that bacterial entry into bursal tissue is partially dependent on T3SS-1. At individual time-points, a significant difference in invasion was observed at 0.5 h (*P* = 0.006) but not at 1 (*P* = 0.224) or 3 h (*P* = 0.064). Notwithstanding the small differences in apparent invasion based upon bacterial counts, which partly reflect the variation between individual infections, there was a clear differences were observed in bacterial location. Wild-type bacteria were concentrated in the bursal FAE (Figure 9A). No invasion of bursal tissues by the Δ*prgH::kan* strain was observed in whole mount images or confocal analysis of tissues (data not shown). Wild-type (Figure 9B), but not the Δ*prgH::kan* strain, were detected in the bursal tissue by 3 h post-inoculation by confocal analysis. *Salmonella* were not detected in the IFE. The vast majority of bacteria detected in the FAE were found within *CSF1R*-transgene negative TIM4^+^ FAE phagocytes and contained within LAMP1+ vesicles, often as large clusters of cells (Figure 9C). Only in rare instances were individual bacteria observed within *CSF1R*-transgene expressing FAE cells (Supplementary Figure 6). In follicles that had been invaded by *Salmonella* the FAE cells showed evidence of blebbing, cell death and shedding into the bursal lumen (Figure 9B; Supplementary Figure 7).

## Discussion

Understanding the biology of antigen uptake in the gut has been fundamental to the study of mucosal immune responses in mammals. Mammalian M cells are the primary cell type that transports microbes and macromolecules across the epithelial cell layer from the gut lumen to the underlying GALT. Some pathogens specifically target M cells to gain entry to host tissues and by mimicking these pathogens the uptake and efficacy of vaccines can be substantially improved by targeting them to M cells (45–47). Mammalian and avian mucosal antigen-sampling cells occupy similar anatomical niches and resemble each other in terms of the uptake of macromolecules, transcytosis of antigens and ultra-structure (3). In mammals FAE enterocytes have the plasticity to transition into M cells (48). This process is dependent on stimulation from the cytokine receptor activator of NF-κB ligand (RANKL) (49) and intrinsic expression of the Ets transcription factor Spi-B (50). However, the *spiB* gene was reported to be absent from the chicken genome (51) and is not currently annotated in any other avian genome. Comparative gene expression profiling of chicken and mouse identified very few genes that were specifically enriched in FAE compared to IFE in common between both species (6). This suggests that, despite functional and morphological similarities, the underlying ontogeny and developmental regulation of mammalian M cells and chicken antigen sampling FAE cells is likely to differ significantly.

To date, no specific markers for the avian equivalent of mammalian M cells in the GI tract have been described. We found that antigen-sampling cells in the bursa and in GALT are tagged by expression of a *CSF1R*-transgene reporter. Retrospective analysis of an earlier independent transcriptomics data set [(6); GEO accession number GSE 16081] also revealed that *CSF1R* expression was enriched in the bursal FAE compared to the IFE. Data in the current study show that in the avian FAE CSF1R is expressed by antigen sampling epithelial cells in addition to macrophages. We confirmed that putative markers of M cells, annexin A10, CD44, L-plastin, (6) as well as EEA1 and gamma-actin, also distinguish cells of the bursa FAE from the IFE. While annexin A10 and EEA-1 were expressed in *CSF1R*-transgene expressing FAE cells in the bursa, neither marker was expressed in the *CSF1R*-transgene expressing antigen sampling cells previously identified in BALT (Supplementary Figure 2B). Therefore, the *CSF1R*-transgene expression is currently the only practical marker for antigen sampling FAE cells in chicken MALT structures.

Contrary to some previous reports, we show that the FAE of the bursa is not a homogeneous structure (6, 52). Staining the bursal FAE with lectins has been previously reported (11, 36, 37). We found that the cells that stain with the lectins jacalin, PHA-L and SNA, lacked expression of CSF1R and have had little antigen uptake capacity. The exact developmental relationship between these two FAE cell populations requires further analysis. The distribution of F-actin in bursal FAE cells is consistent with the role of actin in remodeling of the apical cell surface during active endocytosis and transcytosis (53–55). We demonstrated that the CSF1R protein is expressed by FAE cells, consistent with the transgene expression. The predominant location, on the basolateral membranes and in intracellular vesicles, does not suggest a direct role in antigen uptake. CSF1 action in macrophages is associated with the uptake and destruction of the receptor (56). The presence of the receptor within cytoplasmic vesicles could reflect continued internalization. Using a recently-generated monoclonal antibody, we have confirmed that the ligand, CSF1, is highly-expressed in the bursa (Wu et al., manuscript in preparation). In mammalian macrophages, CSF1R signaling results in the active remodeling of the actin cytoskeleton and rapid morphological changes, including lamellipodial protrusions and membrane ruffling (57). The mechanisms by which CSF1 signaling in macrophages acutely regulates membrane motility and macropinocytosis have been studied is some detail (58–60) and might provide a model for understanding the regulation of antigen uptake in avian FAE. FAE cells that lacked *CSF1R*-transgene expression had reduced cellular F-actin and less apparent involvement in bead transcytosis. Based on the known role in mammalian macrophages and previous evidence that the chicken CSF1R can signal in mouse myeloid cells (61) we suggest that CSF1R is likely to control F-actin dependent remodeling of the cytoskeleton and regulate transcytosis in bursal FAE cells.

Endocytosis of particles by cells can occur via a number of different pathways (62, 63) and particle size can determine the pathway of uptake (64–66). Murine PP and Rabbit appendix M-cells endocytose a broad range size range of particles (67–70), in contrast we show that while bursal FAE cells exhibited efficient uptake of latex beads between 0.02 and 0.1 μm, uptake of bacterial sized ranged 0.5 μm beads was poor. We extended these findings to demonstrate poor uptake of *Salmonella* by the bursal FAE. In these studies, there was substantial divergence between individual birds but entry into the FAE was partially dependent on T3SS-1, which promotes active invasion of enterocytes and M cells in mammals (40). A major difference between chicken bursa FAE and IFE cells was the lack of lysozyme in the FAE. The IFE produces the antimicrobial peptide cathelicidin-B1 (71), suggesting bursal IFE represents a significant barrier to invading microorganisms. It is perhaps unsurprising then that *S*. Typhimurium was found within the bursal FAE, but not the IFE. However, the invasion of bursal FAE by *Salmonella* was not uniform. In some cases, multiple bacteria were being found in individual FAE cells, but despite this, the majority of bursa follicles remain free of bacterial cells, indicating that uptake of bacteria is not a default function of bursa FAE cells. Within the FAE while some individual bacteria were observed within *CSF1R*-transgene expressing FAE cells the vast majority of bacteria were observed within TIM4+ phagocytes and in LAMP+ vacuoles. In mammalian macrophages, *Salmonella* are able to survive within a modified LAMP1+ phagosome known as the *Salmonella*-containing vacuole. *S*. Typhimurium is also able to manipulate the migratory properties of GALT phagocytes to disseminate systemically (72). We suggest that, rather than reflecting a host bacterial killing mechanism, the location of *Salmonella* within LAMP+ FAE vacuoles and TIM4+ FAE phagocytes may aid infection and dissemination of this pathogen in chickens, perhaps explaining the more rapid systemic dissemination of *Salmonella* after intracloacal, rather than oral challenge (73).

The expression of the CSF1R in FAE may provide some insight into the differentiation of these cells. Murine PP M cells are derived from intestinal crypt-based Lgr5^+^ stem cells (48). The differentiation and survival of mammalian M cells depends upon continuous signaling from CSF1R, but receptor expression is restricted to specialized macrophages of the lamina propria that interact intimately with the stem cell niche in the crypts (15). The expression of this receptor clearly distinguishes avian FAE cells from mammalian M cells. By contrast to the expression of *CSF1R* in mammalian trophoblasts, which involves a separate promoter from macrophages, there is no evidence for an alternative promoter in birds (74). The activity of the reporter gene also suggests that macrophages and FAE cells share a transcriptional regulatory mechanism. Embryonic studies are equivocal on whether bursal FAE cells have a mesenchymal or haematopoietic origin (75–77). As CSF1R-mediated signaling is required for the survival, proliferation and function of myeloid cells [(78), review] the expression of CSF1R in bursal FAE cells suggests they are derived from the myeloid cell lineage. In support of this proposal, transcriptome analysis [(6); GEO accession number GSE 16081] indicates that transcripts for transcription factors normally strongly associated with macrophage lineage differentiation and CSF1R transcription (14), such as CSF1R itself, PU.1 and CEBPA, are significantly upregulated in bursal FAE cells compared to IFE cells. However, this may simply reflect the presence of TIM4+ phagocytes in the FAE, as TIM4 gene expression is also enriched in bursal FAE compared to IFE cells (GEO accession number GSE 16081). These data and the present study do not resolve this relationship and further work will be required to determine the relationship between avian CSF1R+ macrophages and the FAE lineages.

In summary, we have shown that the *CSF1R*-transgene reporter chicken can be used to visualize and characterize avian antigen sampling cells. The bursal FAE consists of a heterogeneous population of cells, comprised of epithelial cells which show variable levels of *CSF1R*-transgene expression, F-actin staining and differential expression of carbohydrate moieties, and a population of TIM4^+^ phagocytes which are also found in the bursal medulla. *CSF1R*-transgene expressing FAE cells rapidly take up and transcytose latex beads from the bursal lumen, indicating that this transgene can be used to identify the avian functional equivalent of murine M cells. Given the clear differences between birds and mammals, it is an open question whether these cells should be called M cells. Future studies will resolve the developmental relationship between avian CSF1R^+^ macrophages and CSF1R^+^ antigen sampling FAE cell lineages and determine which growth factors are required to regulate the development and function of these cells.

## Data Availability Statement

All datasets generated for this study are included in the article/Supplementary Material.

## Ethics Statement

CSF1R-eGFP/mApple reporter transgenic chickens and wild type control chickens were supplied at 4–6 weeks of age by the National Avian Research Facility, the Roslin Institute, Edinburgh (UK). The chickens were housed in groups of 22–60 birds and received food and water *ad libitum*. The chickens were maintained under conventional conditions and received standard vaccination scheme against Marek's disease virus, *Eimeria* spp., infectious bronchitis virus, infectious bursal disease virus and Newcastle disease virus. Animals were housed in premises licensed under a UK Home Office Establishment License in full compliance with the requirements of the Animals (Scientific Procedures) Act 1986. Breeding of transgenic chickens was carried out under the authority of Project License PPL 70/8940 with the consent of the Roslin Institute Animal Welfare and Ethical Review Board. Administration of *Salmonella* was undertaken under the authority of Home Office project license PCD70CB48, with the consent of the Ethical Review Committee of the Moredun Research Institute. Chickens inoculated with *Salmonella* were not vaccinated and were housed separately from other birds.

## Author Contributions

AB conceived and carried out the experiments. AB wrote the manuscript with support from DH, NM, HS, LV, and MS. CC-U, PV, RC-C, and MS developed the fluorescently tagged *Salmonella* and helped with infection challenge experiments. TH developed the TIM4 monoclonal antibody and helped with discussions on this part of the project. KS provided the data on the lung and helped with discussions on this part of the project. DD aided in interpreting the results. All authors provided critical feedback and helped shape the research, analysis and manuscript. LV supervised the project.

### Conflict of Interest

The authors declare that the research was conducted in the absence of any commercial or financial relationships that could be construed as a potential conflict of interest.
