# Supplementary material for: Antigen Sampling CSF1R-Expressing Epithelial Cells Are the Functional Equivalents of Mammalian M Cells in the Avian Follicle-Associated Epithelium
Source: Front Immunol. 2019 Oct 22;10:2495. doi: 10.3389/fimmu.2019.02495 (PMC6817575; doi:10.3389/fimmu.2019.02495)
Supplement: Supplementary file 1 [file Presentation_1.PPTX]

## Slide 1
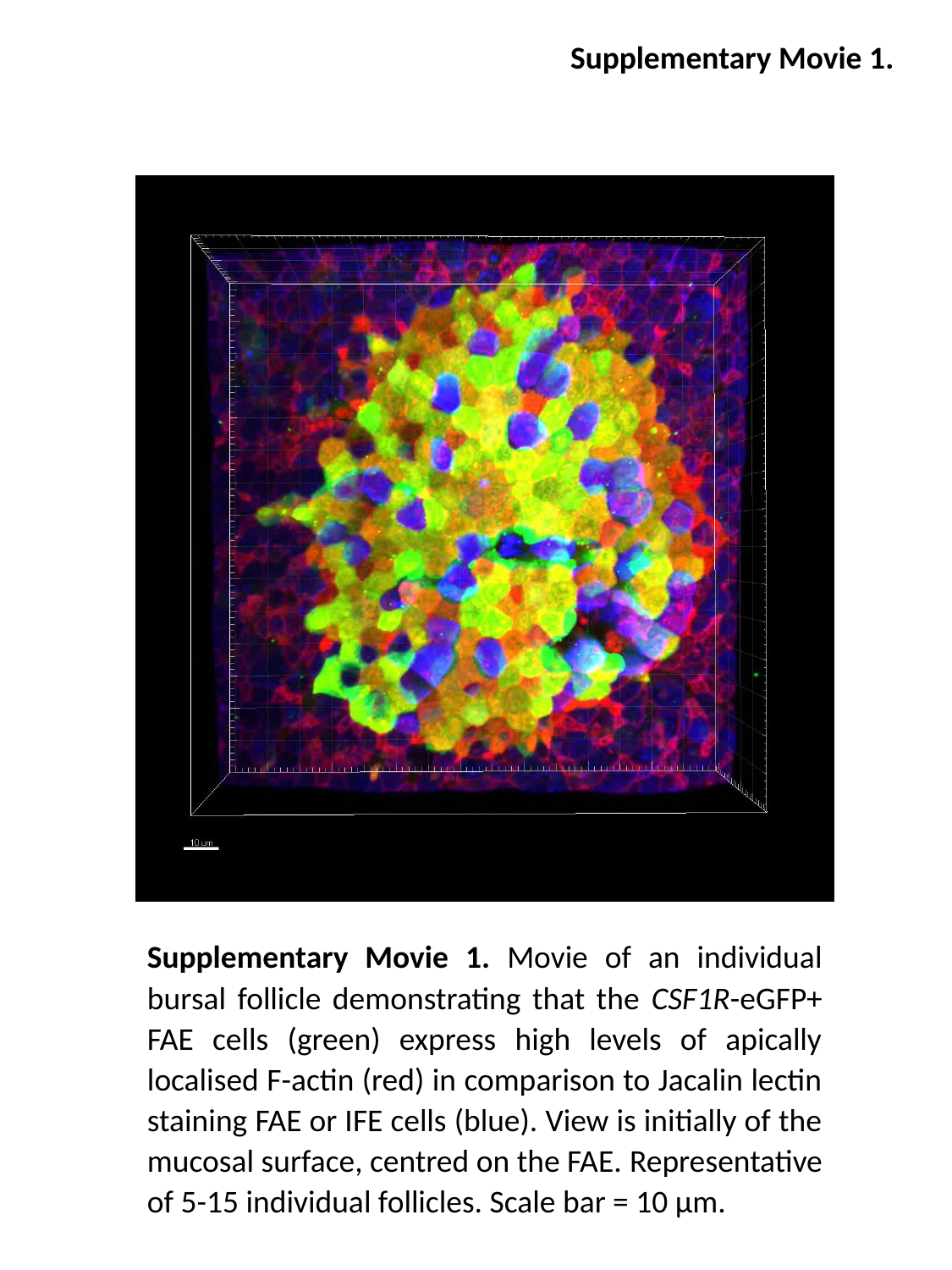

Supplementary Movie 1.
Supplementary Movie 1. Movie of an individual bursal follicle demonstrating that the CSF1R-eGFP+ FAE cells (green) express high levels of apically localised F-actin (red) in comparison to Jacalin lectin staining FAE or IFE cells (blue). View is initially of the mucosal surface, centred on the FAE. Representative of 5-15 individual follicles. Scale bar = 10 µm.
