# Supplementary material for: Antigen Sampling CSF1R-Expressing Epithelial Cells Are the Functional Equivalents of Mammalian M Cells in the Avian Follicle-Associated Epithelium
Source: Front Immunol. 2019 Oct 22;10:2495. doi: 10.3389/fimmu.2019.02495 (PMC6817575; doi:10.3389/fimmu.2019.02495)
Supplement: Supplementary file 2 [file Presentation_2.PPTX]

## Slide 1
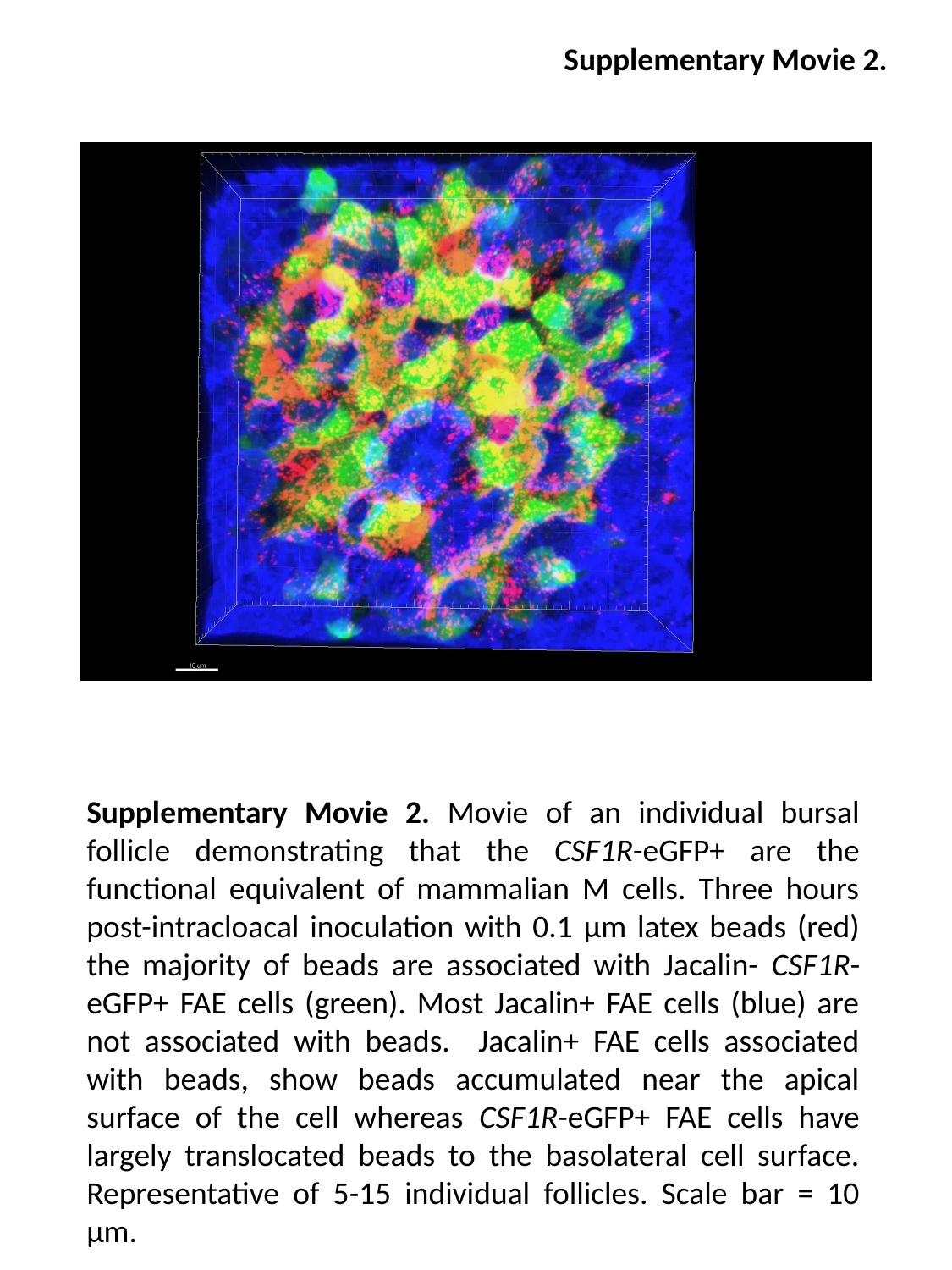

Supplementary Movie 2.
Supplementary Movie 2. Movie of an individual bursal follicle demonstrating that the CSF1R-eGFP+ are the functional equivalent of mammalian M cells. Three hours post-intracloacal inoculation with 0.1 µm latex beads (red) the majority of beads are associated with Jacalin- CSF1R-eGFP+ FAE cells (green). Most Jacalin+ FAE cells (blue) are not associated with beads. Jacalin+ FAE cells associated with beads, show beads accumulated near the apical surface of the cell whereas CSF1R-eGFP+ FAE cells have largely translocated beads to the basolateral cell surface. Representative of 5-15 individual follicles. Scale bar = 10 µm.
