# Supplementary figures and images for: Antigen Sampling CSF1R-Expressing Epithelial Cells Are the Functional Equivalents of Mammalian M Cells in the Avian Follicle-Associated Epithelium
Source: Front Immunol. 2019 Oct 22;10:2495. doi: 10.3389/fimmu.2019.02495 (PMC6817575; doi:10.3389/fimmu.2019.02495)

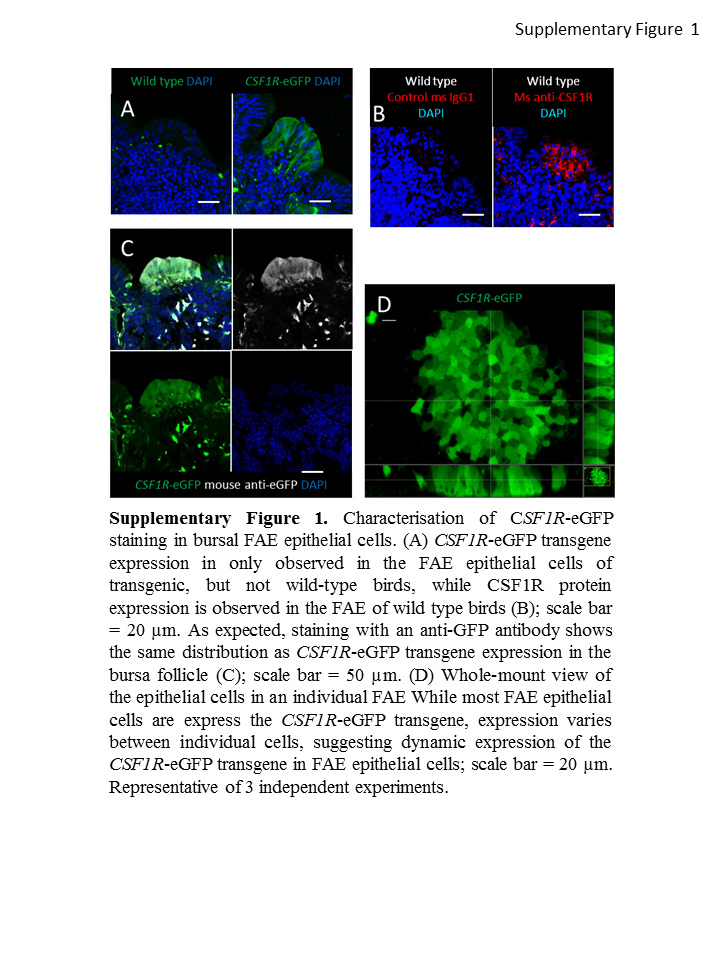

Supplement: Supplementary file 3 [file Image_1.TIF]

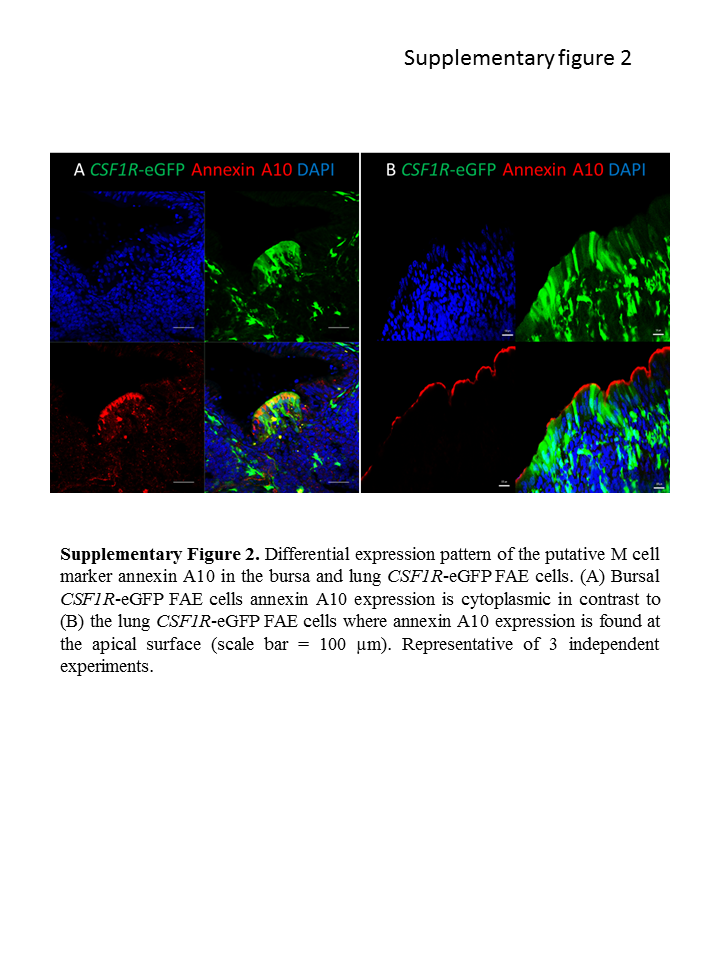

Supplement: Supplementary file 4 [file Image_2.TIF]

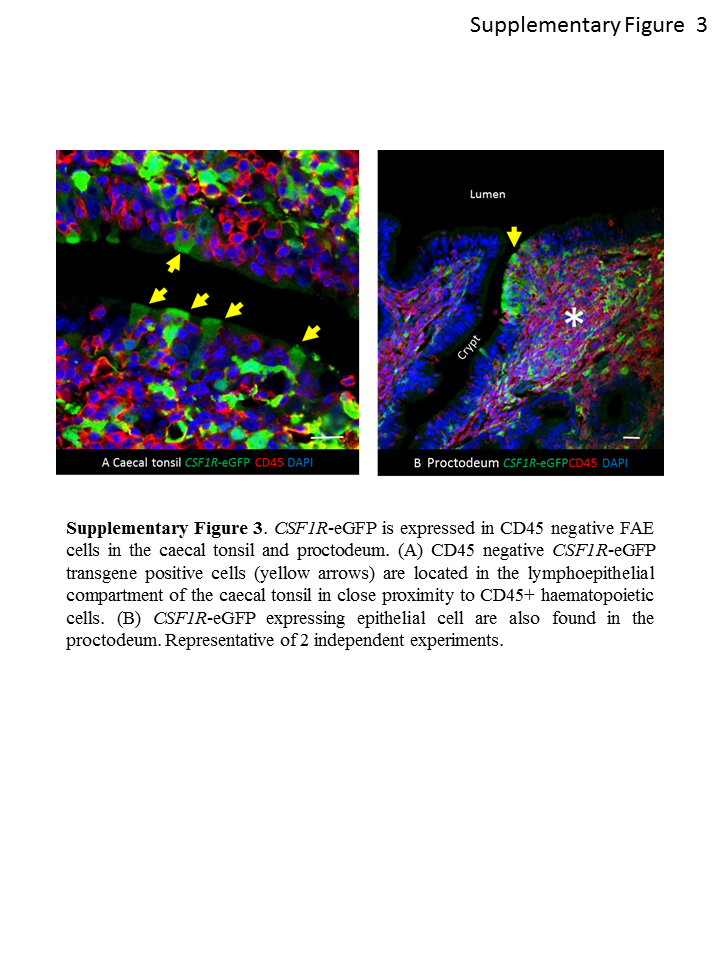

Supplement: Supplementary file 5 [file Image_3.TIF]

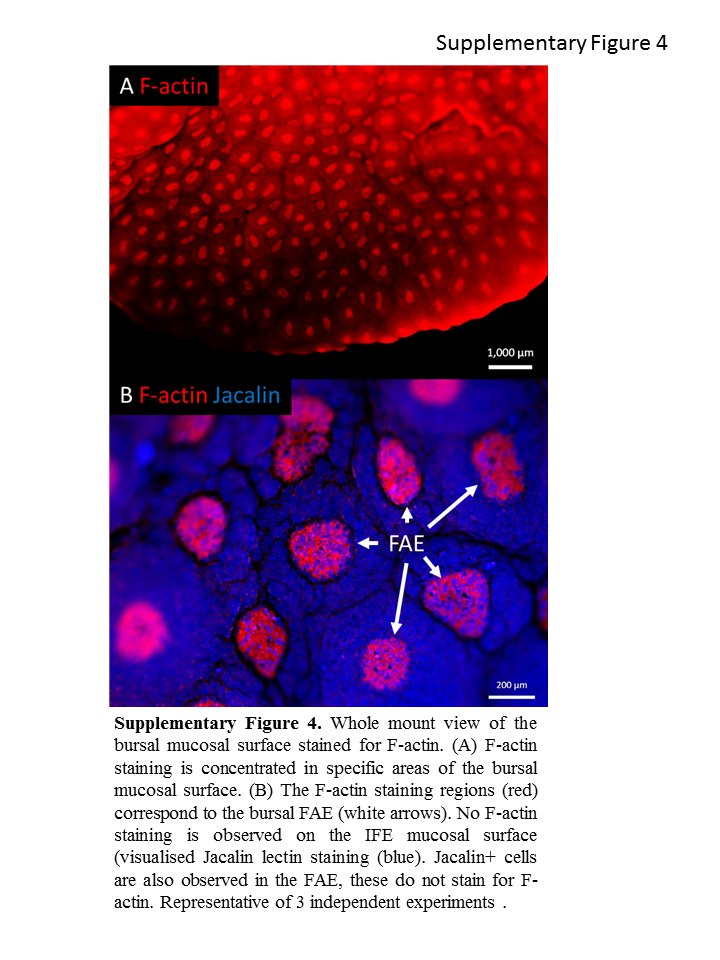

Supplement: Supplementary file 6 [file Image_4.TIF]

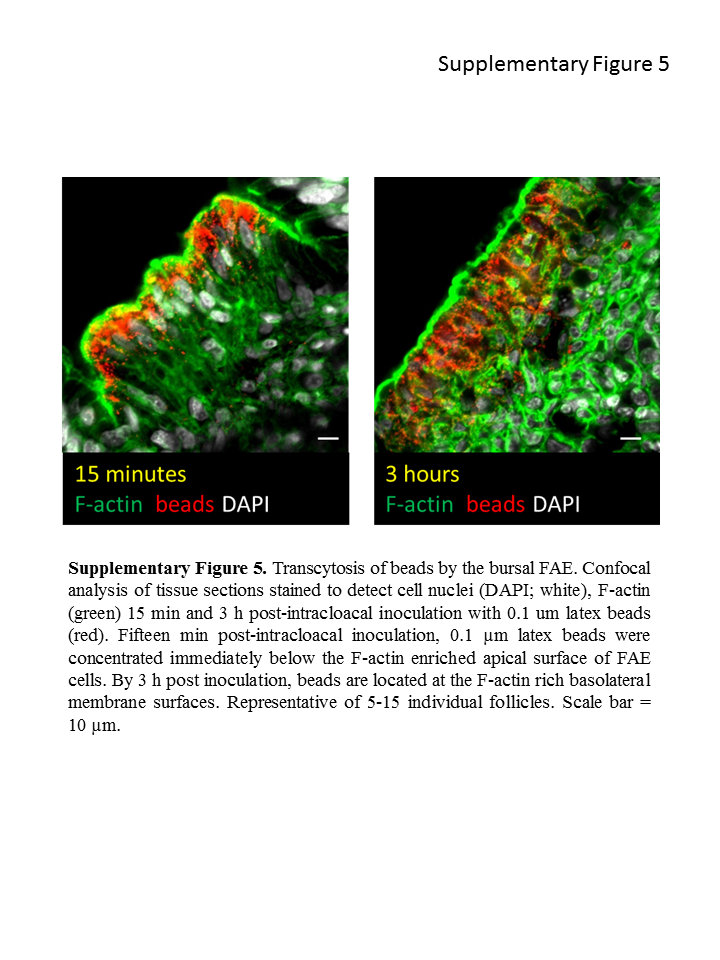

Supplement: Supplementary file 7 [file Image_5.TIF]

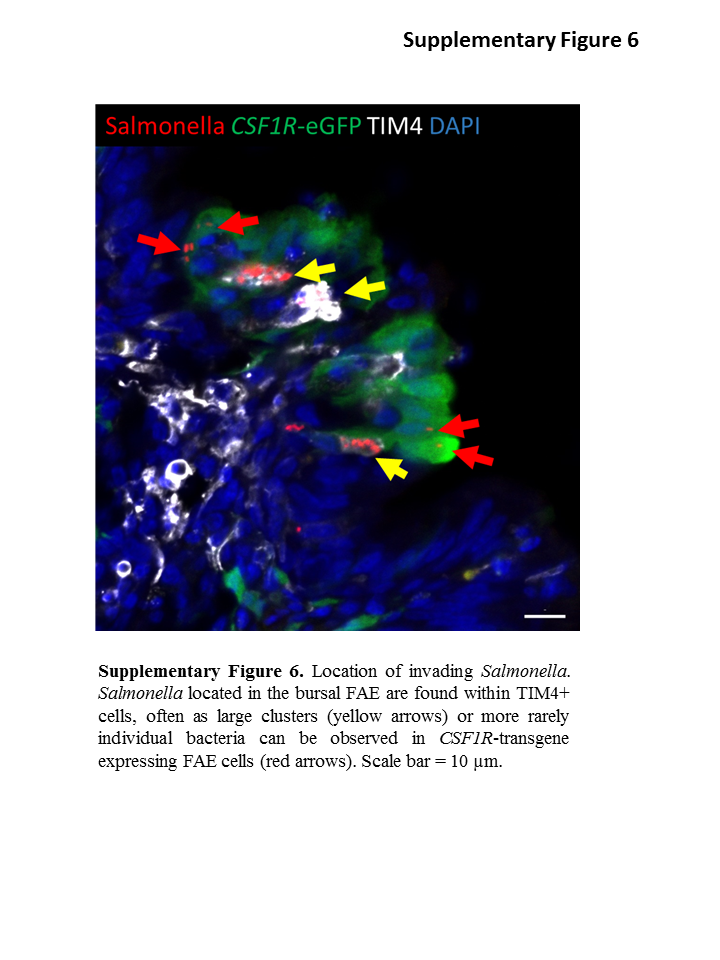

Supplement: Supplementary file 8 [file Image_6.TIF]

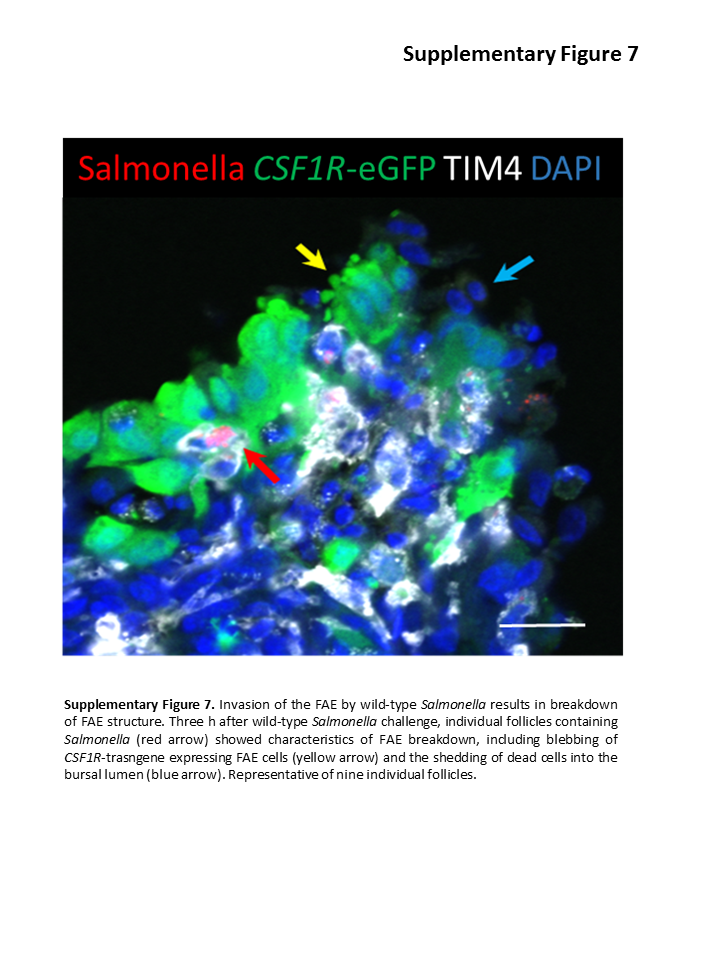

Supplement: Supplementary file 9 [file Image_7.TIF]
